# Effectiveness of platelet-rich fibrin matrix treated with silver nanoparticles in fracture healing in rabbit model

**DOI:** 10.14202/vetworld.2018.944-952

**Published:** 2018-07-16

**Authors:** Serwa Ibrahim Salih, Nadia H. Al-Falahi, Ali H. Saliem, Ahmed N. Abedsalih

**Affiliations:** 1 Department of Surgery and Obstetrics, University of Baghdad, Baghdad, Iraq; 2 Department of Physiology, Biochemistry and Pharmacology, University of Baghdad, Baghdad, Iraq

**Keywords:** bone gap, healing, platelet-rich fibrin matrix, silver nanoparticles

## Abstract

**Aim:**

The current study was conducted to evaluate the effect of platelet-rich fibrin matrix (PRFM) treated with silver nanoparticles (AgNPs) on enhancing the healing of the experimentally induced bone gap in a rabbit model.

**Materials and Methods:**

Twenty healthy male local rabbits aged between 6 and 8 months, their weights between 1.5 and 2 kg were used in this study and divided randomly into four equal groups, under general anesthesia (1 cm), bone gap was induced in the tibia bone to create a critical bone defect and leave it without any treatment in the first group (control group). While in the second group the bone gap was filled with PRFM; in the third group, the gap was filled with 0.3 ml AgNPs; and in the fourth group, the gap was filled with PRFM treated with AgNPs.

**Results:**

There was no infection at the operation site in all experimental animals, and the radiograph images showed periosteal and endosteal reaction; the gaps were bridged faster in the fourth group as compared with the other groups. The histological examination showed lamellar bone with haversian canal completely filled the fracture gap and contact with old bone in the fourth group as compared to other groups.

**Conclusion:**

Using a combination of PRFM and single nucleotide polymorphisms together gave better acceleration in the bone healing process than using each one of them separately.

## Introduction

Bone repair is a complex process that involved cellular functions and mineralization followed by remodeling of the defect site to attain the original structure [1]. Several studies have shown that bone regenerative procedures may be accelerated by the addition of specific growth factors [2,3]. Platelet-rich fibrin (PRF) represents a simplified processing and artificial biochemical modification in the platelet gel therapeutic conception [4]. The preparation process of PRF matrix (PRFM) creates a gel-like matrix which is characterized by high levels of non-activated, functional, intact platelets and trapped within a fibrin matrix that releases a relatively constant concentration of growth factors over a period of 7 days [5-7]. Nanotechnologists have become participates in regenerative medicine by the creation of nanostructures and biomaterials with potential clinical implications, aiming at developing systems that can create and reinforce *in vivo* tissue repair strategies [8].

Nanomaterials had been developed quickly because of their exact characteristics such as size and morphology [9]. These properties related to grander specific areas and energies, which normally increased the reactivity of surface than those of the grander particles, leads to greatly changed properties. Due to these features, nanoparticles (NPs) were increasingly used in many products such as energies, medicines, environmental remediation, and biomedical devices [10-12]. Among these NPs, silver NPs (AgNPs) had gained attention due to their exclusive biological, chemical, and physical properties in comparison to their large size equivalents [13,14]. Nanosilver was evidenced to be a most active material which possesses respectable antibacterial properties against different microorganisms, for example, viruses, bacteria, fungi, and parasite [15].

The aim of current study was to evaluate the combination of PRFM with AgNPs on tibia bone gap repair in rabbits.

## Materials and Methods

### Ethical approval

The animal utilization protocol of the experiment was approved by the animal care and use committee with approval number 1647 in 25-04-2016, College of Veterinary Medicine, University of Baghdad, Baghdad, Iraq.

### Biological preparation and characterization of AgNPs

Fresh olives leave collected from local olive trees in Baghdad. Then washed with water and left to dry at room temperature for about 1 month, and pounded to a fine powder by an electrical processor. Olive leave powder grouping was conducted in the state board for seeds testing and certification in Baghdad. The aqueous extraction of olive leaves was made by putting 50 g of prepared powder in 500 ml of sterile D.W, the mixture then heated for 10 min until the mixture color become faint yellow. At that point, the obtained extract cooled and filtered at room temperature [16].

The preparation of AgNPs was made by mixing 100 ml of 10³M AgNO3 solution with 5 ml of olive leaves extract with stirring to get a faint yellow solved at room temperature. The blend heated in a water bath at 40 and 60°C. Changing in color of the blend was monitored at different temperatures and times as explained here: 40°C 5 min reaction time, 60°C 10 min reaction time, and 60°C 15 min reaction time. The AgNPs obtained by centrifugation of the blend at 15,000 rpm for 10 min then the sediment re-disposed in sterile D.W. to eliminate uncoordinated materials [16].

### Characterization of AgNPs

#### Spectrophotometer

Ultraviolet (UV)-visible spectrophotometer was used to study the optical features of biologically synthesized AgNPs. This apparatus was used to confirm creation and constancy of AgNPs in sterile D.W. in wavelength ranged from 200 to 800 nm. The reduction of Ag+ to Ag° was checked by spectrophotometer after mixing the extract with an AgNO3 solution, and the measuring was at regular periods from 0 to 15 min through taking 1 ml from each solution [17].

#### Scanning electron microscope (SEM)

The shape and size of synthesized AgNPs were examined using SEM by dropping the very small amount of the sample on the specialized grid (carbon coated copper) [18].

#### Preparation of PRFM

PRFM is prepared in our laboratory according to Dohan *et al*. [4] to produce PRF membrane which used in the present study. 3 ml of blood sample was collected by cardiac puncture without anticoagulant in glass tubes, then immediately centrifuged at 3000 rpm for 10 min, three separated layers were resulted: The lower layer represented the red blood corpuscles, PRFM as a fibrin clot was in the middle, while acellular platelet poor plasma was the superficial layer. The matrix was withdrawn with forceps from the tube, cutting off the red blood corpuscles, then squeezing of PRFM from the fluid between two metal slides to obtain a very resistant autologous fibrin membrane, which was trimmed in a piece size of approximately 0.5 cm×1 cm [4].

#### Experimental design

Twenty healthy male local rabbits aged between 6 and 8 months, their weights between 1.5 and 2 kg were used in this study and divided randomly into four equal groups:

First group (control group): 1 cm bone gap was induced in the shaft of tibia and left to heal normally without additives.
Second group (PRFM group): The same operation was followed, but the bone gap was filled with PRFM.
Third group (AgNPs group): The same operation was followed, but the bone gap was filled with 0.3 ml AgNPs solution.
Fourth group (PRFM and AgNPs group): The same operation was followed, but the bone gap was filled with the PRF membrane (not gel or liquid) treated with 0.3 ml AgNPs, so there are no changes in the dose of AgNPs which used in present study.

### Surgical procedure

The animals were injected with Acepromazine maleate (10 mg/kg BW.) I/M as a tranquillizer and after 10 min a mixture of ketamine hydrochloride (35 mg/kg BW.) and xylazine (5 mg/kg BW.) was injected I/M [19], the operation site was surgically prepared, the animal cast laterally and a surgical incision (3 cm) were made at the midshaft of the tibia from the medial aspect, the subcutaneous tissue was opened then the bone was exposed (1 cm), bone gap was created using an electric drill to create a critical bone gap, then washed with normal saline and filled with PRFM or AgNPs or both of them in treated groups, while in control group the gap was left without additives, then the muscles were sutured with PDS (3-0), the skin was sutured with simple interrupted suture using surgical silk (3-0). All animals were given a systemic antibiotic for 5 days (penicillin 10000 IU/kg BW. and streptomycin 10 mg/kg BW. I/M).

All animals were exposed to radiography after 2, 4, 6, and 8 weeks post-operation and euthanized after 2 and 4 weeks post-operation; the specimens were analyzed for the histopathological examination to notice the osteogenesis at the site of the fracture.

## Results

### Biological preparation of AgNPs

**Figure-1:**
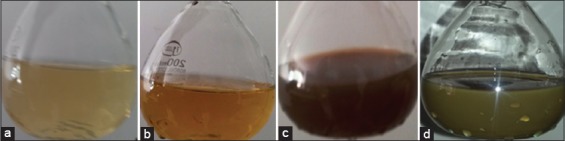
Changing in color during the preparation of silver-nanoparticles (a) pale yellow color when olive leaves extract was mixed with the silver nitrate solution. (b) Deep yellow color at 40°C after 5 min of reaction time. (c) Deep brown color at 60°C after 10 min of reaction time. (d) Gray-black color at 60°C after 15 min of reaction time.

The current study provided evidence that the olive leaves were a good source for synthesizing stable AgNPs in lesser time. The total amount of AgNPs which produced from 500 g of olive leaves powder was 17.5 g. When olive leaves extract was mixed with the AgNO3 solution at room temperature gave pale yellow color, after 5 min reaction time at 40°C the color of the solution altered from faint yellow to profound or deep yellow color at 40°C and 5 min reaction time; this alteration in color indicating of AgNPs formation due to a decrease in Ag+. When the temperature of water bath increased to 60°C for 10 min, the color of blend altered to deep brown color, and finally the color of the mixture became gray-black at 60°C after 15 min reaction time (Figure-1).

### Characterization of AgNPs Spectrophotometer

**Figure-2:**
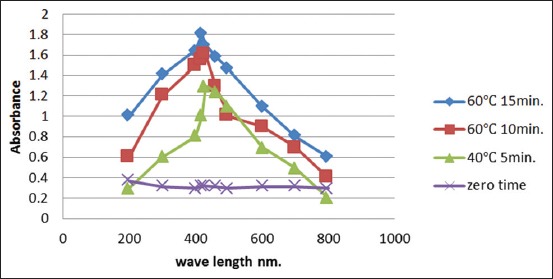
Ultraviolet-visible absorption spectra of silver nanoparticles synthesized by olive leaves extract at different times and temperatures.

The resulted color of mixing olive leaves extract with the AgNO3 solution was pale or faint yellow color, examination the mixture at this time which considered zero time by UV-vis spectra revealed no sign and no peak for the preparation of AgNPs. At 40°C after 5 min reaction time, the color altered from faint yellow and UV-visible spectra gave surface plasmon resonance (SPR) for synthesized AgNPs which produced a peak centered at 420 nm matched to the absorbance of AgNPs. Furthermore, at 60°C after 10 min of the reaction time and 60°C after 15 min of the reaction time the peak of the absorbance became at 430 nm and 420 nm respectively (Figure-2).

### SEM

**Figure-3:**
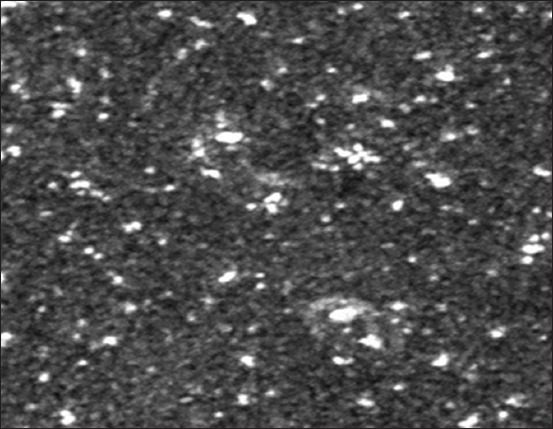
Scanning electron microscope image shows the synthesis of silver nanoparticles with mean diameter 26 nm with the relatively spherical shape.

SEM gave further understanding of the shape and size details of the synthesized AgNPs. The outcomes demonstrated that the mean diameter of produced AgNPs was around 26 nm. The image also showed relatively spherical shaped NPs (Figure-3).

### Clinical findings

All experimental animals appeared healthy, without any complications (infection or rejection) in the site of operation, but the animals were unable to bear weight on the operated limb for about 3 days then the animals stood on their forelimb normally.

### Radiological findings

#### In the first group (control group)

**Figure-4:**
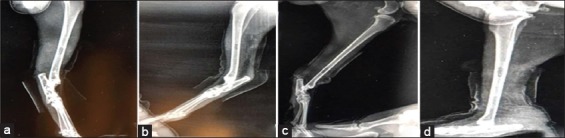
Bone defects immediately post-operation (a) control group, (b) second group, (c) third group, and (d) fourth group.

**Figure-5:**
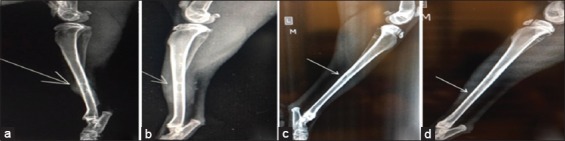
Bone defects at 2 weeks post-operation (a) control group, (b) second group, (c) third group, and (d) fourth group.

**Figure-6:**
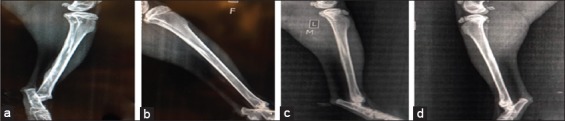
Bone defects at 4 weeks post-operation (a) control group, (b) second group, (c) third group, and (d) fourth group.

**Figure-7:**
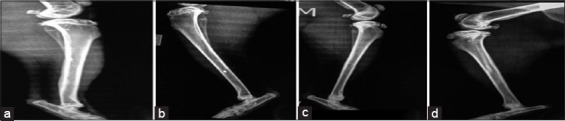
Bone defects at 6 weeks post-operation (a) control group, (b) second group, (c) third group, and (d) fourth group.

**Figure-8:**
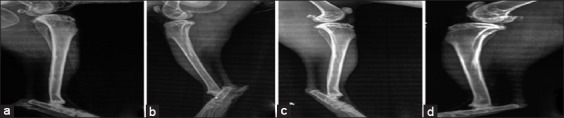
Bone defects at 8 weeks post-operation (a) control group, (b) second group, (c) third group, and (d) fourth group.

The radiograph showed the created defect in the tibia (radiolucent area) immediately postoperatively (Figure-4a). After 2 weeks post-operation, the radiograph showed a clear defect with signs of periosteal and endosteal reactions at the peripheries (Figure-5a). At 4^th^-week post-operation, the bone gap still can be seen, not closed yet which appeared as a radiolucent area (Figure-6a). At 6^th^-week post-operation, the tibia defect can be observed (Figure-7a). At 8^th^-week post-operation, the radiograph showed that the defect could be observed as a radiolucent slit (Figure-8a).

#### In the second group (PRFM)

The radiograph showed the created defect in the tibia filled with PRFM (radiolucent area) postoperatively (Figure-4b). At 2^nd^-week post-operation, the radiograph showed the gap with slight radio-opacity (Figure-5b). At 4^th^-week post-operation, the radiograph showed that the gap could be observed as a thin radiolucent area (Figure-6b). At 6^th^-week post-operation, the radiograph showed a minimized defect that barley could be observed (Figure-7b). At 8^th^-week post-operation, the radiograph showed the tibia with clear medullary canal and normal structure no sign of the defect (Figure-8b).

#### In the third group (AgNP)

The radiograph showed the created defect in the tibia treated with AgNPs (radiolucent area) postoperatively (Figure-4c). At 2^nd^-week post-operation, the radiograph showed that the gap is still clear (radiolucent) at this period (Figure-5c). At 4^th^-week post-operation, the radiograph showed that the gap could be observed as a thin radiolucent area (Figure-6c). At 6^th^-week post-operation, the radiograph showed that the gap could be observed smaller (Figure-7c). At 8^th^-week post-operation, the radiograph showed the tibia with irregular cortices, medullary canal not clear but the defect cannot be observed (Figure-8c).

#### In the fourth group (PRFM with AgNP)

The radiograph showed the created defect in the tibia treated with PRFM and AgNP, (slightly radiopaque area) postoperatively (Figure-4d). At 2^nd^-week post-operation, the radiograph showed that the defect could be observed with radio-opacity, reflecting periosteal reaction within (Figure-5d). At 4^th^-week post-operation, the radiograph showed clear radiopaque area, bone cortices irregular, thickened, and medullary canal not clear (Figure-6d). At 6^th^-week post-operation, the radiograph showed barely seen defect (Figure-7d). At 8^th^-week post-operation, the radiograph showed clear medullary canal with no signs of any defect (Figure-8d).

### Histopathological evaluation

#### In the first group (control group)

**Figure-9:**
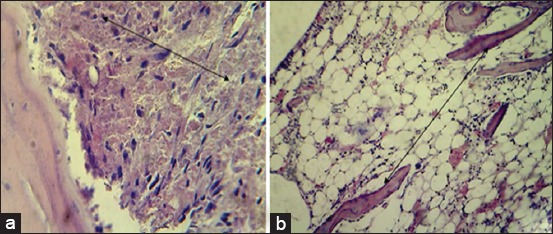
Histopathological section in control group at 2 weeks post-operation, (a) shows red blood cells and inflammatory cells in bone gap(arrow) (b) shows bone trabeculae lined by osteoblast extended into narrow cavity(arrow) (H and E, 100×).

**Figure-10:**
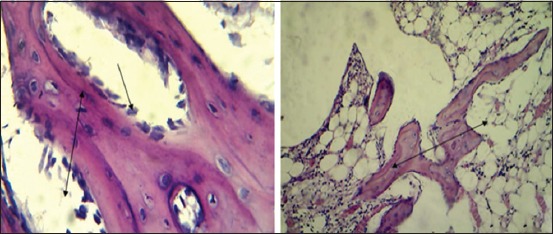
Histopathological section in control group at 4 weeks post-operation, (a) shows lamellar bone containing wide haversian canal (double head arrow) lining by active osteoblast in gap fracture (arrow), (b) shows moderate thickness trabecular bone in the bone gap (arrow) (H and E, 100×).

The histopathological findings after 2 weeks post-operation showed cellular debris, dead neutrophils red blood cell (RBCs), and inflammatory cells in the gap (Figure-9a) and the bone trabeculae lining by osteoblast extended into marrow cavity (Figure-9b). At 4^th^-week post-operation, lamellar bone containing wide haversian canal lining by active osteoblast (Figure-10a), with moderate thickness trabecular bone was observed in the gap (Figure-10b).

#### In the second group (PRFM)

**Figure-11:**
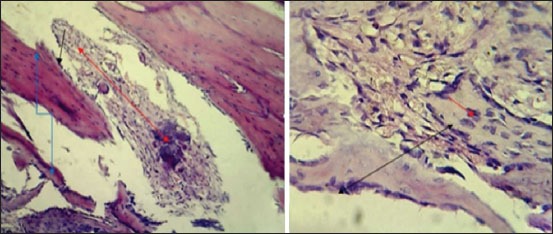
Histopathological section in the second group at 2 weeks post-operation, (a) shows proliferation of fibrous connective tissue (red arrow) and moderate thickness trabecular bone (blue arrow) lined by osteoblast in the fracture gap (black arrow) (H and E, 100×), (b) shows thick trabecular bone lined by active osteoblast (black arrow) with large osteocytes (red arrow) extended from each side of the gap (H and E, 400×).

After 2 weeks post operation, the histopathological findings showed proliferation of fibrous connective tissue with moderate thickened trabecular bone lined by osteoblast in the gap (Figure-11a) with presence of inflammatory cells, RBCs with thick trabecular bone lined by osteoblast with large osteocytes extended from each side of the bone gap (Figure-11b)

**Figure-12:**
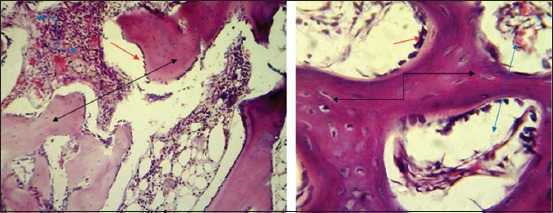
Histopathological section in the second group at 4 weeks post-operation, (a) shows thick trabecular bone (black arrow) lined by active osteoblast (red arrow) extended from each side that filled the gap with round cells infiltration between large spaces between these trabecular bones (blue arrow) (H and E, 100×), (b) shows the gap filled by moderate thickness trabecular bone (black arrow) form variety size HCs (blue arrow) that lined by active osteoblast (red arrow) (H and E, 400×).

After 4 weeks post operation, the section**s** showed thickened trabecular bone lining by osteoblast extended from each side that filled the gap cavity with round cells infiltration between large spaces between these trabecular bones (Figure-12a) and the gap filled by moderate thick trabecular bone, forming various sizes of haversian canal lined by osteoblast (Figure-12b).

#### In the third group (AgNP)

**Figure-13:**
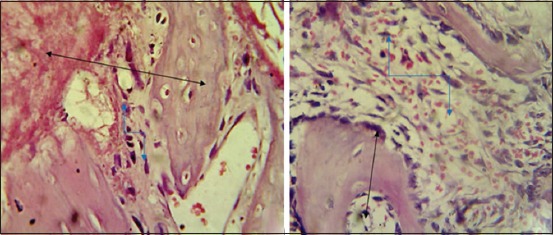
Histopathological section in the third group at 2 weeks post-operation, (a) shows fibrin networks and trabecular bone extended into the gap (black arrow) with inflammatory cells infiltration (blue arrow), (b) shows thick trabecular bone lined by marked active osteoblasts around large spaces (black arrow) with round inflammatory cells and proliferation of fibrous connective tissue around necrotic bone fragment in the gap (blue arrow) (H and E, 400×).

**Figure-14:**
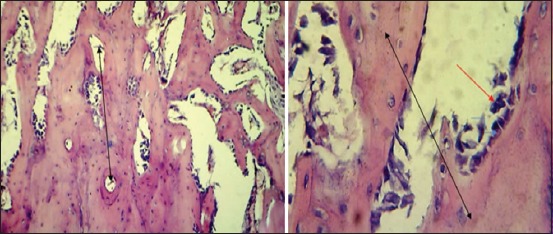
Histopathological section in the third group at 4 weeks post-operation, (a) shows lamellar bone containing large size and number of osteocytes with variable size of haversian canal lining by active osteoblasts extended from each side of the gap (arrow), (b) shows laminar with large size and number of osteocytes (black arrow) with variable size of haversian canal lining by active osteoblasts with osteoclasts extended from each side of the gap (red arrow) (H and E, 100×).

The histopathological findings after 2 weeks post-operation showed fibrin networks and trabecular bone extending into the gap with infiltration of inflammatory cells (Figure-13a). Thick trabecular bone lined by marked active osteoblasts around large spaces with round inflammatory cells and proliferation of fibrous connective tissue was observed in the gap (Figure-13b). At 4^th^-week post operation, the section**s** showed lamellar bone containing large number and size of osteocytes with variable size of haversian canal lining by osteoblasts extended from each side of the bone gap (Figure-14a). Other sections showed lamellar bone containing large number of osteocyte with variable size of haversian canal lining by osteoblasts with osteoclasts which extended from each side of the bone gap (Figure-14b).

#### In the fourth group (PRFM with AgNP)

**Figure-15:**
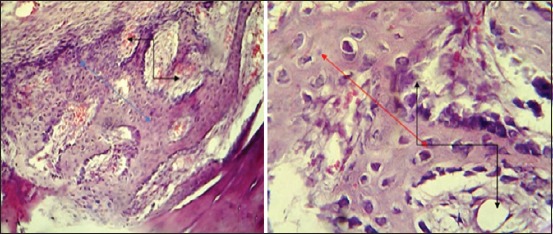
Histopathological section in the fourth group at 2 weeks post-operation, (a) shows woven bone consisting from irregular trabecular bone (blue arrow) with large number of osteoblasts and surrounded numerous variable size spaces (black arrow) (H and E, 100×), (b) shows large number of osteocytes (red arrow) and numerous variable size spaces lining by active osteoblasts (black arrow) (H and E, 400×).

**Figure-16:**
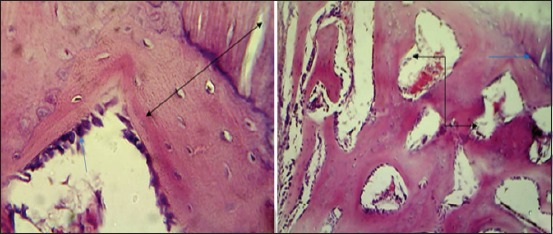
Histopathological section in the fourth group at 4 weeks post-operation, (a) shows newly synthesis compact bone with haversian canal lining by active osteoblasts attachment with old one filled the gap (arrow), (b) shows laminar bone with HC completely filled the fracture gap (black arrow) and contact with old bone (blue arrow) (H and E, 400×).

After 2 weeks post-operation The histopathological findings showed woven bone consisting of irregular trabecular bone with many osteoblasts and surrounded by numerous variable size spaces (Figure-15a). Woven bone consisting of irregular trabecular bone with many osteocytes and surrounded by numerous variable size spaces lined by osteoblasts was seen (Figure-15b). At ^4th^-week post operation, the sections showed newly synthesized compact bone with haversian canal lining by osteoblasts attached with tibia bone filled the gap (Figure-16a). Lamellar bone with haversian canal completely filled the bone gap and connected with the tibia bone (Figure-16b).

## Discussion

### Biological preparation of AgNPs

This study provided evidence that the olive leaves were a good source for synthesizing stable AgNPs in less time. When olive leaves extract was mixed with the AgNO3 solution, the color of the solution changed from pale yellow color to deep yellow color at 40°C and 5 min of reaction time. This change in color regard to the reduction of silver ions indicating AgNPs formation. The mechanism by which the plant extract could be synthesized AgNPs may be indicated by the higher total content of phenols and flavonoids [20,21].

Oleuropein is the major phenolic component of olive leaves. These phenols and flavonoids have high reducing capacity which leads to the AgNPs formation [22]. These NPs exhibited yellowish brown color in aqueous solution due to excitation of surface plasmon vibrations in AgNPs, and this result has been previously obtained by several investigators [23,24]. Increasing the temperature of water bath to 60°C and 10 min of reaction time changed the color of the mixture to deep brown, and at 15 min of reaction time at 60°C, the deep brown color changed to gray-black; this further color change was due to increased concentration of NPs with assisting of temperature which increases reduction rate and formation of NPs [25].

### Characterization of AgNPs

#### Spectrophotometer

AgNPs are sensitive to shape; size, concentration, and agglomeration state and vigorously interact with specific wavelengths of light. This strong interaction with light occurred due to the conduction electrons on the metal surface undergoes a collective oscillation when they were excited by light at specific wavelengths and this oscillation is known as an SPR, and it causes the scattering and absorption intensities of AgNPs to be much higher than identically sized non-plasmonic NPs, so at zero time, UV-visible spectra showed no peak because the AgNPs not formed yet, i.e., there was no any concentration of AgNPs, whereas at 5, 10, and 15 min after reaction, there was an increase in absorbance due to increasing NPs concentration, and the UV-visible spectra showed peak at 420 nm and 430 nm, which belong to the absorbance of AgNPs. This result agreed with several studies showed that the AgNP surface plasmon vibrations peak at around 420 nm [23,26]. These peaks appeared single and narrow which means that the biological preparation of AgNPs was stable and did not aggregate. Scattering from a tested sample of AgNPs was very sensitive to the aggregation instance of the sample, where the scattering increases when the particle aggregate to a large extent. When particles aggregate and the conduction electrons near each particle surface become delocalized and are shared among neighboring particles, this leads to the change of the optical properties of AgNPs. When this occurs, the SPR shifts to lower energies, causing the absorption peak to redshift to longer wavelengths. Hence, the UV-visible spectroscopy can be used as a simple and reliable method for monitoring the stability of NP solutions. As the particles destabilize, the original extinction peak will decrease in intensity (due to the depletion of stable NPs.), and often the peak will broaden or a secondary peak will form at longer wavelengths (due to the formation of aggregates) [27]. However, AgNPs with negative surface charge and this high negative charge of particles increase its stability (more than 1 year) due to repulsion between the particles that avoid its aggregation [28].

#### SEM

AgNPs were circular or spherical shape; smooth edges were observed under an electron microscope. The distribution particle size marginally varied with the variations in duration time. The particle numbers elevated with increasing duration due to the change in the reducing process. These results are in agreement with the observation that recorded by other researches [28].

#### Bone radiography and histopathology

In fracture, the inflammation is equivalent to other tissue responses to injury. However, during the early days development of the primary callus occurs, which appears to be a basic reaction to bone injury. This first callus response is poorly organized and composed of calcified cartilage and woven bone, ultimately remodeled into a mechanically competent bone structure. The biological events in fracture repair are finite, and an overshoot in fibroblast tissue regeneration may induce a shift from competent calcified tissue to incompetent fibrous tissue. PRFM is an autologous fibrin matrix, which trapped a large quantity of platelet and leukocyte cytokines through centrifugation [29]. The intrinsic integrate of cytokines within the fibrin matrix permits for their progressive release over a period of 7-11 days, as the network of fibrin disintegrates [30]. The easily applied PRF matrix acts as a fibrin bandage [31], serving in acceleration the healing process [32]. It also provides good protection of the operation site and seems to enhance the integration and remodeling of the grafted biomaterial [29]. Radiographical finding of the effect of PRFM in second and fourth groups at different intervals postoperatively showed that the process of ossification started after 2 weeks and filled with osseous tissue after 4 weeks; this result agrees with Dohan *et a*l. [5], who found that the utilize of PRF had reduced the time of healing and brought a faster bone regeneration. Meanwhile, bone radiography in the third- and fourth-treated groups showed that the bone returned to the normal structure after treatment with single-nucleotide polymorphisms (SNPs). Some researchers indicated that biomaterials are commonly applied in regenerative therapy and tissue engineering in bone. Local application of NPs may be useful for many potential uses with the improvement of tissue regeneration and enhanced osseointegration of implants with the prevention of infections [33]. Other researchers exhibited that the SNPs accelerate fracture healing and induced early closure of the bone gap by promoting bone callus formation; the subsequent fracture ends joining of by chemoattraction of the mesenchymal stem cells [34]. Results of histological finding showed that using of autologous PRFM in the induced gap has benefits for organizing the formative cell (especially osteoblast), formation of neovascularization and more rapid and faster apposition of the bony matrix with its mineralization process. These results were supported by the increase in number of trabecular bone, osteoblasts, osteocytes, and blood vessels in comparison with control group, this may regard to the ability of PRFM to integrate with fibrin network and facilitates cellular migration especially endothelial cell which recorded high number of blood vessels formation (neoangiogenesis) in comparison to control group, more supplementation of blood to healing area accelerate and potentiate two processes: First process involved self-regulation of inflammatory and infectious phenomena by leukocytes and cytokines which present in fibrin matrix and blood. Second process includes providence of nourishment for undifferentiated cells to be differentiated and provides significant effects for their migration to the healing area and activates its biological role [30]. The increase in numbers of osteoblasts (in treated group) there will be more trapped osteoblasts in an osteoid matrix which appear as the increment in osteocyte number and consequences to the osteoid formation, trabeculae width will leading to decrease in trabecular separation in comparison to control group. The result of this study revealed that there is increase in trabecular and cortical bone width and osteoclast number as the experimental period progress and that is true on the fact that osteoid formation leads to bone trabeculae formation, apposition, maturation and thickness with time. The effect of the PRFM appeared in enhancement for cell migration and endothelial cell formation. Fibroblasts migrate to the site of fracture; inducted the proliferation and osteogenesis of mesenchymal stem cells or induction the differentiation of osteogenic mesenchymal stem cells through induction/activation of transforming growth factor-beta/bone morphogenetic protein (BMP) signaling in mesenchymal stem cells. In addition, some authors indicated that the NPs stimulate and promote the differentiation, mineralization of osteoblast and increase bone density by stimulation of the autophagy [35]. On the other hand, some researchers showed that the NPs support the osteoregeneration by the direct influence of osteoblast differentiation and osteoclast behavior [33]. Others revealed that the SNPs induce bone ingrowth, cell migration, tissue ingrowth, and vascularization. Mammalian macrophages have ability to sense peptidoglycan [36-38]. The macrophages have a great role in the expression of inflammatory cytokines and growth factors [39]. Therefore, it has a central role in fracture healing [40]. However, the induction of inflammatory cytokines and fibrosis enhancing growth factors by stimulated macrophages suggests a prominent osteolytic effect as the macrophages lost the ability to synthesize BMP-2 under pro-inflammatory conditions [41]. On this basis, some authors suggested that osseous healing is damped by conditions that enhance the proinflammatory activity of the macrophage [40].

## Conclusion

The current study indicated that using a combination of PRFM and SNPs together gave better acceleration in the bone healing process than using each one of them separately.

## Authors’ Contributions

SIS and NHA contributed to the planning and doing research work as study design, and writing. AHS and ANA contributed to the pharmacological aspects of the research. All authors read and approved the final manuscript.
